# Monitoring serum HER2 levels in breast cancer patients

**DOI:** 10.1186/s40064-015-1015-6

**Published:** 2015-05-22

**Authors:** Julia Tchou, Lian Lam, Yun Rose Li, Claire Edwards, Bonnie Ky, Hongtao Zhang

**Affiliations:** Division of Endocrine and Oncologic Surgery, Rena Rowan Breast Center, Abramson Cancer Center, Perelman School of Medicine, University of Pennsylvania, Philadelphia, PA 19104 USA; Department of Pathology and Lab Medicine, University of Pennsylvania Perelman School of Medicine, 3620 Hamilton Walk, Philadelphia, PA 19104 USA; Medical Scientist Training Program; Perelman School of Medicine, University of Pennsylvania, Philadelphia, PA USA; The Center for Applied Genomics, The Children’s Hospital of Philadelphia, Philadelphia, PA USA; Division of Cardiovascular Medicine, Perelman School of Medicine, University of Pennsylvania, Philadelphia, PA 19104 USA

**Keywords:** HER2/neu, SHER2, Biomarker, MBB buffer, Breast cancer

## Abstract

**Background:**

We have developed a new approach to reduce the serum interference for ELISA. The purpose of this study is to investigate if we can use the optimized ELISA (MBB-ELISA) to detect serum soluble HER2/neu (sHER2) in early stage primary breast cancer and monitor its change during treatments.

**Findings:**

We collected sera preoperatively from 118 primary breast cancer patients. Serum samples were also collected sequentially from a subset of patients during and after adjuvant treatment. sHER2 in these samples was measured by the MBB-ELISA. Only 16.7 % of tissue HER2 (tHER2) positive patients had significantly elevated sHER2 levels in serum. Interestingly, sera of some patients with tHER2 negative tumors, including those that were 2+ by IHC but negative by FISH, demonstrated slightly elevated sHER2 levels. Multivariate analysis demonstrated that patients with elevated sHER2 (> = 7 ng/ml) had significantly worse disease free survival. During treatments, sHER2 levels consistently fell in response to adjuvant therapies. Nevertheless, in all 4 patients who developed metastases, a steady rise in sHER2 levels was noted before metastatic disease became clinically evident.

**Conclusions:**

For early stage breast cancers, sHER2 is a poor biomarker to predict tHER2 status, but may have value to supplement tissue tests to identify patients with HER2 tumors. Our results also suggest that sHER2 is worth further study as a biomarker to monitor breast cancer patients during treatments.

## Findings

### Introduction

Currently, women with breast cancer are eligible for HER2/neu (HER2) based therapy if their tumors express the HER2 protein, as determined by immunohistochemistry (IHC) staining, or if their tumors demonstrate *her2/neu* gene amplification, as determined by fluorescence in situ histochemistry (FISH). In addition to trastuzumab, several other HER2/neu based targeted therapies are now available, including pertuzumab and the antibody-drug conjugate ado-trastuzumab emtansine (Slamon et al. 2001; Zhang et al. 2007; Franklin et al. 2004; Cai et al. 2008; Barok et al. 2011). However, IHC and FISH analyses have to be performed on tumor tissues, which may not always be available. In addition, heterogeneous expression of HER2/neu has been reported in breast cancer at both the cellular and tissue level and may result in false negative HER2/neu expression status (Potts et al. 2012). An alternative method to ascertain tumor HER2/neu expression status that does not rely on tissue sampling would be of value to guide treatment strategy for patients (Lam et al. 2012).

One such alternative method is to measure serum levels of the extracellular domain (ECD) portion of the HER2/neu receptor. During tumor development, tumor cells shed the ECD of HER2/neu (also known as soluble HER2, or sHER2) into the circulation (Tse et al. 2012; Lam et al. 2012) by proteolytic cleavage. ADAM10 was identified as one of the critical metalloproteinases responsible for the cleavage of HER2/neu (Liu et al. 2006; Christianson et al. 1998). The presence of circulating or soluble HER2 ECD (sHER2) has made it possible to develop serum or plasma-based tests for HER2 (Zhang et al. 2001; Zhang et al. 2006).

Although FDA-approved tests for sHER2 measurement are commercially available, these tests are not broadly used in clinical settings as they are susceptible to high background signals (Lam et al. 2012) which are often caused by the presence of human anti-animal immunoglubulins (HAIA) in the serum (Papoian 1992). We have developed a method to eliminate these HAIA using a special MBB buffer (Zhang et al. 2011). Using this approach, we studied serum sHER2 by using the mouse antibody mAb 4D5 to capture sHER2 in the sandwich enzyme-linked immunosorbent assay (ELISA) (Lam et al. 2014). As the therapeutic antibody trastuzumab was derived from mAb 4D5, this assay can potentially interfere with trastuzumab in the serum. To circumvent this potential interference, we have adapted our platform by utilizing a new set of antibodies that recognizes unique epitopes distinct from trastuzumab, in order to detect sHER2 from serum samples of both trastuzumab naïve and treated patients (Lam et al. 2012).

In this study, to study the correlation between sHER2 and tissue HER2 (tHER2) status, we first compared tested sHER2 and tHER2 in 118 patients undergoing primary breast cancer surgery. Because a subset of these patients with tHER2 positive disease also had serum samples collected at various time points during and after their adjuvant therapy, we also sought to determine the changes in sHER2 over the course of adjuvant therapies and whether sHER2 levels were associated with disease progression. Overall, our results suggest that sHER2 may provide clinical value to monitor the treatment of patients with Her2+ disease.

## Materials and methods

### Patients and serum specimens

Under institutional review board (IRB) approved protocol, we prospectively enrolled breast cancer patients undergoing breast cancer surgery at our institution. The first patient cohort consisted of 118 breast cancer patients treated at our institution between 2010 and 2014. Serum samples were collected preoperatively on the day of their breast cancer surgery after the patients signed the informed consent form. In a subset of these patients, serum samples were collected at standardized intervals during adjuvant treatment.

#### MBB ELISA for HER2 ECD

Anti-HER2 monoclonal antibodies 6E2 and biotinylated A21G were used as the capture and detection antibody, respectively. HER2 ECD standard was originally purchased from Oncogene Sciences. For ELISA, the capture antibody (6E2, 0.2 μg/ml, 50 μL) was suspended in PBS and used to coat a 96-well plate by overnight incubation at 4 °C. After wash with 0.1 % Tween 20 in PBS (PBST), the plate was blocked with 5% bovine serum albumin solution in PBS for 1 h at room temperature (22 °C). HER2 ECD standard and serum samples were diluted in the Magic Blocking Buffer (MBB) (Zhang et al. 2011) and incubated for 1-h at 22 °C. Diluted biotinylated detection antibody (50 μL, 0.5 μg/mL, with MBB) was added to each well for a 1 h-incubation at room temperature. Streptavidin-conjugated horseradish peroxidase (HRP) (R&D systems) was used as the secondary antibody to detect the antigen-antibody complex. The plate was washed three times with PBST in-between incubations. Following six washes with PBST to remove excess detection antibodies, 50 μL of tetramethyl benzidine (TMB) substrate (0.1 mg/mL, 0.05 M phosphate-citrate buffer, pH 5.0) was added to each well at 22 °C. The reaction was stopped within 15–30 min with 50 μL of 2 M H_2_SO_4_, and the data was collected at 450 nm (absorbance filter) and 630 nM (reference filter) using the SpectraFluor reader (Tecan). For comparison, a commercial sHER2 detection kit from Wilex (Wilex/oncogene Science, Cambridge, Mass) was used, and the test was performed according to the recommended procedure.

## Statistical analyses

Demographics and clinical characteristics including race, age at diagnosis (defined as the date of initial breast cancer surgery), tumor subtypes according to receptor status, tumor size (as continuous variable or as categorical variable by Tumor Node Metastasis (TNM) classification), number of involved or (+) axillary lymph nodes (as ordinal or as categorical variable by TNM classification), and types of adjuvant systemic therapy (if applicable) were collected by detailed chart review. Tumor receptor subtypes were classified into three groups: Group 1 was comprised of ER+ breast cancer which expressed either ER or PR and lacks HER2 expression; Group 2 was comprised of HER2+ breast cancer which expressed HER2 as determined by IHC and/or FISH with or without expression of ER or PR; and Group 3 was comprised of TNBC which lacked expression of ER, PR, and HER2. Clinical characteristics and sHER2 measurements were compared across the three groups using either a two-tailed student’s *t*-test for continuous covariates (i.e., age and tumor size) or a two-tailed Fisher’s Exact Test for discrete or ordinal covariates (i.e., histologic types, and number of (+) axillary lymph nodes (0 vs. ≥ 1). Univariate and multivariate analyses were performed to assess both overall and disease-free survival outcomes using Kaplan-Meier analyses and Cox-proportional hazards models.

## Result

### Concordance between MBB HER2 ELISA and the commercial Wilex HER2/neu ELISA assay

Using human HER2-expressing murine cell line T6-17, we have developed several anti-HER2 antibodies (Zhang et al. 2006). Two antibodies, 6E2 and A21G, were selected as a pair of antibodies for the sHER2 ELISA. Using this pair of antibodies and also the MBB buffer (Zhang et al. 2011), we assembled the MBB HER2 ELISA assay to detect sHER2 in serum from patients. Previously, we have shown that the MBB buffer reduced the serum interference and thus eliminated false positive in control samples (Zhang et al. 2011). A critical question to address was whether the MBB affected the readouts for true positive samples. A comparison between the MBB assay and the commercially available Wilex HER2/neu assay (Nuclea Diagnostic, Cambridge, MA) was thus planned using a subset samples (*n* = 40) from our cohort.

**Fig. 1:**
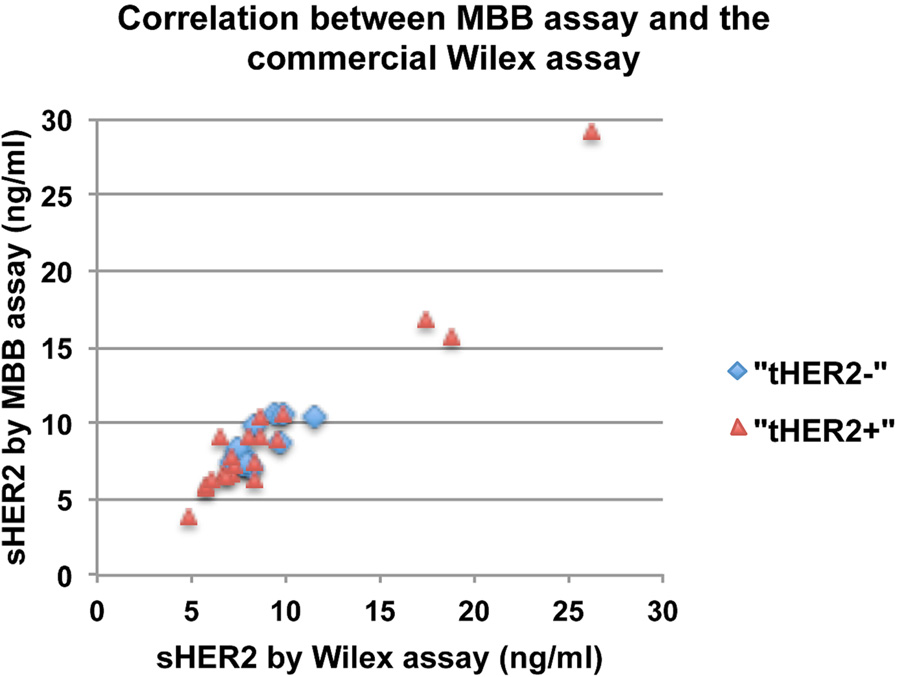
Correlation between MBB assay and the commercial Wilex assay for serum sHER. Forty serum samples were grouped into either tHER2 negative (blue diamond) or positive (red triangle) and tested by both assays. Both groups showed good correlation of sHER2 levels between these two assays

As showed in Fig. 1, sHER2 levels as measured by the two methods showed very similar values. The overall correlation (R^2^) for all samples tested was 0.96307. When samples were grouped according to tissue HER2 (tHER2) status, the two assays appeared to have better correlation in tHER2^+^ samples (R^2^ 0.93952 vs 0.6127 in tHER2-). The weaker correlation in tHER2^−^ samples was likely due to the variability in sHER2 readouts when the target protein was at low abundance. Both assays revealed that tHER2 positive patients could have normal levels of sHER2.

### Correlation of serum levels with tissue HER2 status

**Fig. 2:**
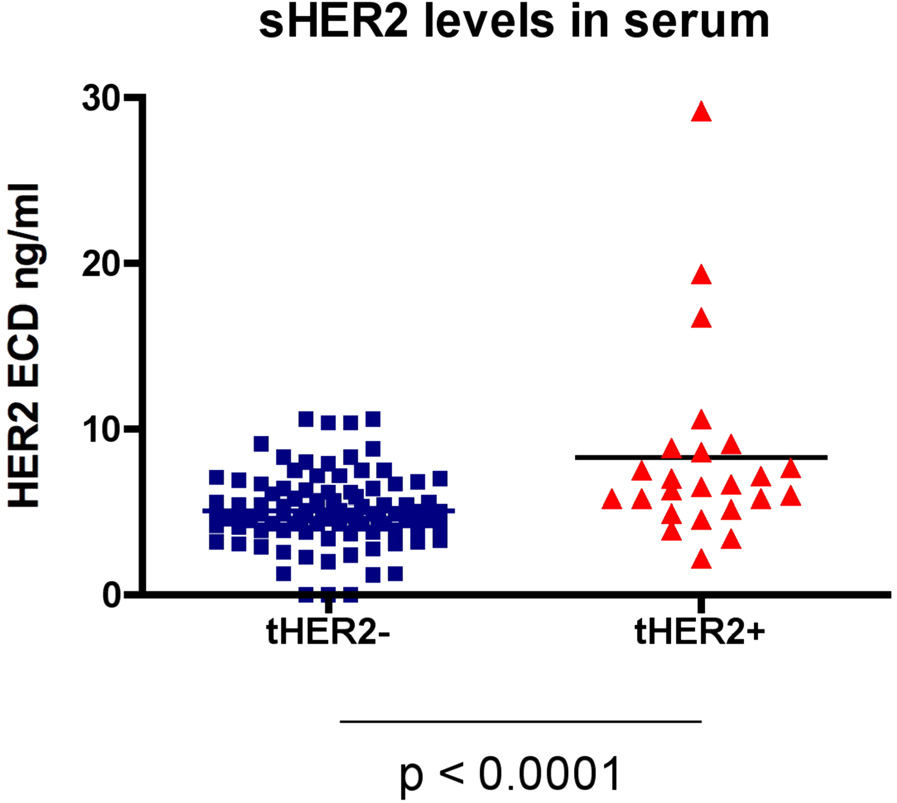
Elevated sHER2 levels in tissue HER2 positive patients (tHER2+). sHER2 levels were determined by MBB ELISA. tHER2+ patients had significantly higher average sHER2 levels

As results of breast cancer serum samples using our own MBB sHER2 assay were comparable to those obtained using the commercially available Wilex assay, we proceeded to use our MBB assay to measure sHER for the rest of our study. The mean sHER2 values and corresponding clinical and tumor characteristics of our study cohort were summarized in Table 1. No significant correlation was noted between sHER2 with age at diagnosis, tumor size, or axilla lymph nodes status. sHER2 levels were significantly different among patients with different races, receptor subtypes or disease status (p-values of 0.04, 0.032 and 0.007, respectively). Interestingly, patients noted to be of Asian descent had significantly higher sHER2 levels compared to those of other ethnicities. As expected, sHER2 levels were significantly higher in tHER2^+^ subtype compared to tHER2^−^ or TNBC subtypes. The sHER2 levels of tHER2^+^ patients ranged from 2.2 to 29.2 ng/ml with a mean of 8.3 ng/ml while the sHER2 levels in tHER2^−^ patients ranged from 0 to 10.4 ng/ml with a mean of 5.1 ± 2.2 ng/ml (Fig 2, *p* < 0.0001). While all of tHER2^−^ patients had relatively low levels of sHER2, it was also evident that only a small fraction of tHER2^+^ patients (*n* = 3) had dramatically elevated serum levels (>15 ng/ml, Fig. 2). Of the 23 patients with tHER2^+^ tumor, 45.8 % had elevated sHER2 levels (> = 7 ng/ml), as defined by sHER2 ’values greater than the 75^th^ percentile, while only 16.7 % (*n* = 16) of tHER2^−^ patients had elevated sHER2 levels (range: 7.0–10.6 ng/ml). Three of these patients were FISH negative but had an IHC score of 2+, indicating a likelihood of HER2 protein overexpression even in the absence of gene amplification.

**Table 1:** Patient characteristics and baseline sHER2 levels

		N	sHer2 values	p values ^a^
	Patients (N)	118	5.750 +/− 3.495	
Race	*Asian*	7	9.11	0.041
	*Caucasian*	81	5.23	
	*African American*	30	6.36	
Age at diagnosis(years)	*mean ± sd*		54.10 +/−13.81	0.813
	*≤45*	32	5.38	
	*>45*	86	5.89	
Tumor size (cm)	*mean ± sd*		2.8- +/− 2.33	.852
T1	*<2*	53	5.49	
T2	*2-5*	53	5.81	
T3	*>5*	11	6.27	
	*N/A*	1		
(+) axilla lymph node(s)	*0*	63	5.47	1.000
	*≥1*	55	6.07	
Receptor subtype	*tHer2+*	23	8.18	.032
	*tHer2-, ER/PR+*	77	5.02	
	*TNBC*	18	5.79	
Clinical Outcome	*Disease Present*	12	8.50	0.007
	*Disease Absent*	106	5.44	
Length of Follow Up (Months)	*Mean ± sd*	23.51 +/− 11.38		
	*Median*	21.32		

Abbreviations: *ER* estrogen receptor, *PR* progesterone receptor, *sHER2*, soluble human epidermal growth factor receptor 2. ^a^This P value was determined using the Fisher’s test. N/A: in 1 patient, relevant information (e.g., tumor size) was missing

### sHER2 levels may have prognostic significance: higher sHER2 levels correlates with worse clinical outcomes

**Fig. 3:**
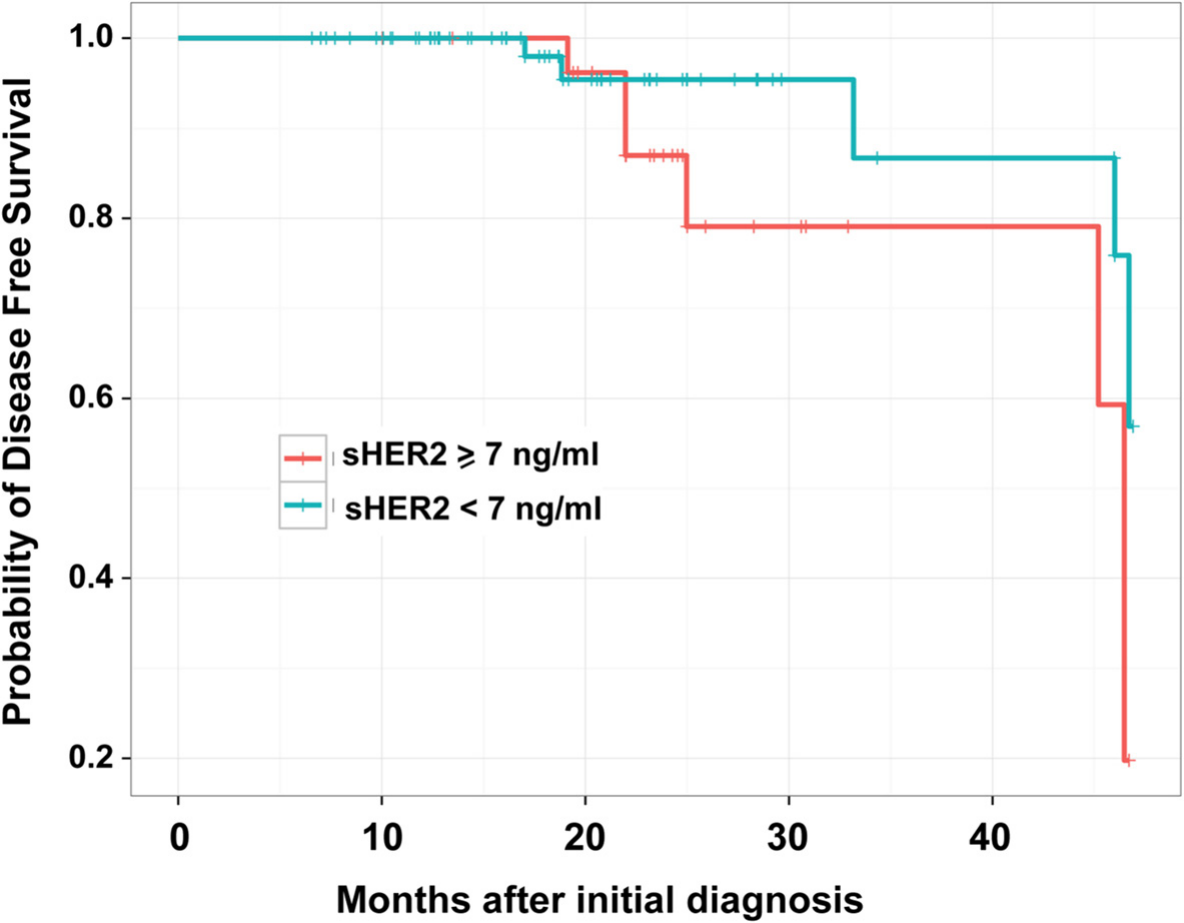
Disease free survival of breast cancer patients after surgery. Kaplan-Meier analyses showing the association of preoperative sHER2 with adverse patient outcome in two cohorts using recurrence-free survival as clinical endpoints

Over a median follow-up of 19 months, we observed disease recurrence in 12 patients (10 %) (Table 1). Patients who developed disease recurrence had significantly higher baseline sHER2 levels than those who remained disease-free (8.5 vs. 5.4 ng/ml, *p* = 0.007, Table 1). To further evaluate if baseline (preoperative) sHER2 correlated with clinical outcomes, Cox analysis was performed to compare patients with sHER2 in the 75^th^ percentile (≥7 ng/ml) and those with lower sHER2 (<7 ng/ml). Univariate analysis demonstrated that sHER2 levels were significantly associated with disease-free survival (log-rank score test: *p* = 0.009, hazard ratio HR = 1.117 +/− 0.044). In multivariate analysis, sHER2 levels remained independently associated with disease-free survival. We further performed Kaplan-Meier analysis (Fig. 3). Disease-free survival was significantly worse in patients with higher sHER levels ((≥7 ng/ml, log-rank score test, *p* = 0.0098).

### Serial sHER2 levels to assess disease progression

**Fig. 4:**
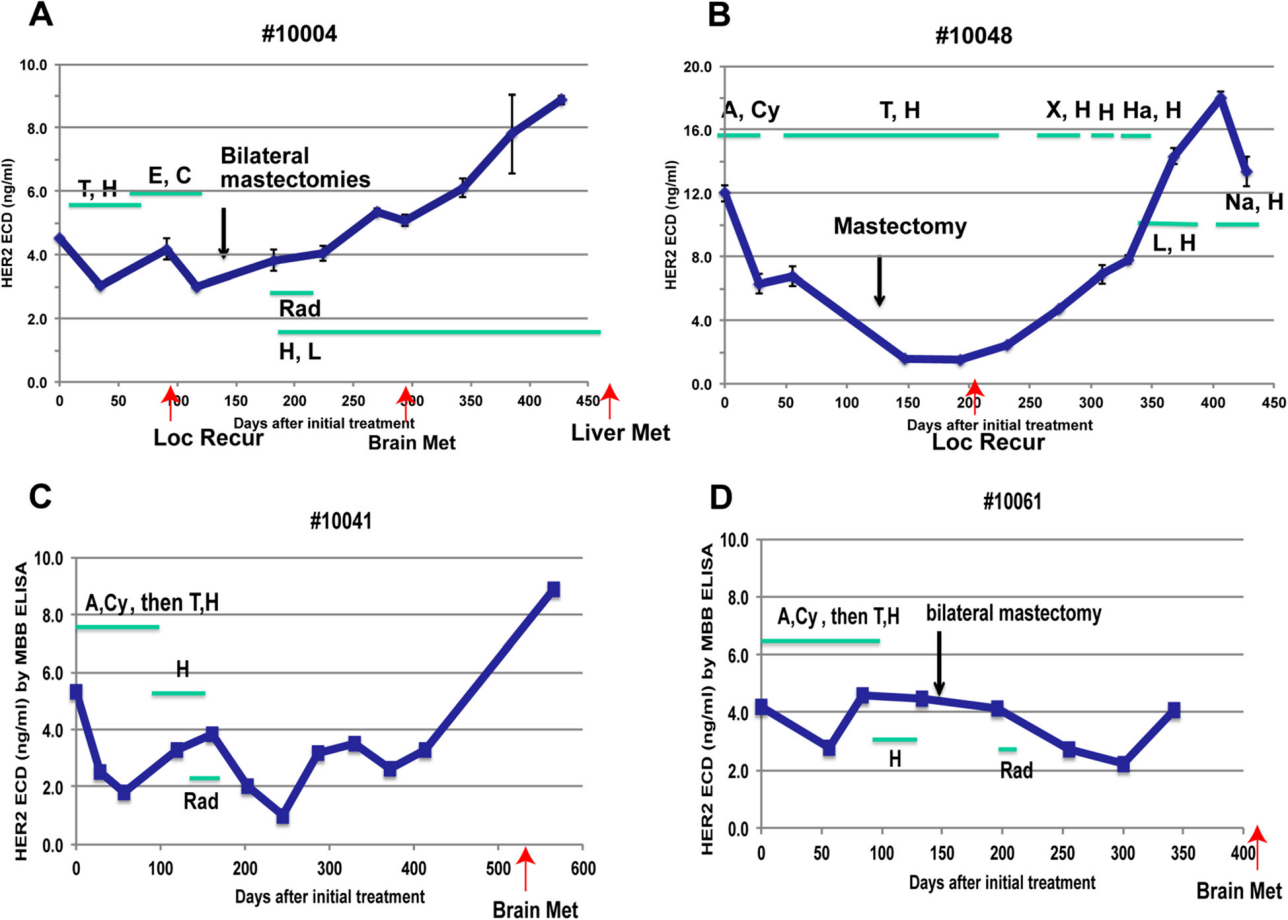
Monitoring sHER2 levels in 4 patients who eventually developed recurred diseases. Serum samples of these patients were collected periodically and tested using MBB-HER2 ELISA. Treatments and recurrence / metastasis were indicated. **a** Patient#10004 failed to respond to adjuvant therapies and had a local recurrence. After bilateral mastectomy, sHER steadily went up, even in the presence of radiation and additional Herceptin treatments. Brain metastasis was identified during the sHER2 rising phase, and eventually the patient developed liver metastasis. **b** Patient#10048 showed a drop of sHER2 after chemo/Herceptin treatments and mastectomy. After about 2 months of staying at the bottom, sHER2 gradually went up after local recurrence was identified. The patient was treated with several kinds of chemotherapies with Herceptin, but sHER2 kept surging. Eventually the navelbine/Herceptin combination was able to change the course of rising sHER2 levels. **c** In patient #10041, sHER2 responded to the initial chemo/Herceptin treatment and dropped to < 2 ng/ml. The level rebounded to ~ 4 ng/ml during the Herceptin alone treatment. After radiation, the level was reduced again to a very low level (<1 ng/ml). However, sHER2 level rose again and brain metastasis was identified. **d** sHER2 levels in #10061 was restricted in a very narrow range over the course of treatment. After bilateral mastectomy and radiation, sHER2 gradually bottomed out at 2 ng/ml. In less than 2 months, sHER2 rose from 2 ng/ml to 4 ng/ml. Two months later, brain metastasis was detected. Serum samples right before metastasis was not available and it is not clear if the upward trend of sHER2 continued until brain metastasis. Treatments: A: Adriamycin; Cy: Cytoxan; T: Taxol; H: Herceptin; X: Xeloda; Ha: Halaven; L: Lapatinib; Na: Navelbine; Rad: radiation

In a subset of patients (*n* = 16), serum samples were collected at various time points during and after adjuvant therapy. Of these 16 patients, four developed disease recurrence. Their serial sHER2 levels over the follow-up period were examined and grafted with their treatment and disease status (Fig. 4). In one patient (#10048), we noted that sHER2 level was significantly reduced after mastectomy. In general, we observed a steady rise in sHER2 levels before distant metastases became clinically evident in three of the four patients who developed disease recurrence (patient 10004, 10041 and 10061).

## Discussions

### Correlation of sHER2 and tHER2 status – sHER2 is not a useful predictive marker for tHER2 positive status in primary breast cancer patients

The ability to measure sHER2 as a non-invasive means to monitor tumor HER2 status has generated much interest in the past 20 years (Colomer et al. 2000; Esteva et al. 2005). Although the sHER2 test has failed to prevail as a standard clinical screening test to determine eligibility of HER2 targeted therapies, many studies have shown the correlation of sHER2 levels with tissue HER2 status, disease stages, and disease free survival (Ludovini et al. 2008). As noted above, one of the major limitations in the serum ELISA assay is the serum interference associated with human anti-animal antibodies that leads to mostly false positive results (Lam et al. 2012). To circumvent this problem, we have developed a unique MBB blocking buffer as described in our earlier studies (Zhang et al. 2011; Lam et al. 2014). In this study, we further evaluated the association between tHER2 and preoperative sHER2 values in a prospective study cohort using our MBB sHER2 assay. We found that only 16.7% of tHER2 positive early stage breast cancer patients had dramatically elevated sHER2 levels.

Our results are similar to those from larger clinical trials involving early stage primary breast cancer patients. In one study, only 12 % of 2318 tHER2 positive patients had elevated preoperative sHER2 levels (Moreno-Aspitia et al. 2013). In another recent study of 2862 cases of stage I–III primary breast cancer patients, 24 % were found to be tHER2 positive, and only 15 % of these tissue positive patients had elevated sHER2 (Lee et al. 2014). Therefore, sHER2 is a poor diagnostic marker for early stage breast cancer and should not be used to replace conventional IHC or FISH analyses to guide treatment planning.

### sHER2 levels correlates with disease status-possible role of sHER2 as a non-invasive biomarker to monitor disease progression during or after adjuvant therapy

In a previous study when we examined a cohort of invasive breast cancer patients that included late stage metastatic cases, elevated sHER2 levels were detected in about 32% of samples, suggesting that sHER2 may be more closely associated with tHER2 positivity in patients with heavier tumor burden or with metastatic disease (Lam et al. 2014). Furthermore, we have previously monitored sHER2 in 12 patients with DCIS who had received dendritic cell-based vaccines against HER2 peptide prior to their definitive surgery (Lam et al. 2014). In one of these 12 patients, sHER2 continued to rise. Metastatic tHER2 positive disease was subsequently diagnosed in this patient. Results from our sequential measurement of sHER2 in patients during and after adjuvant therapy also demonstrated that sHER2 levels would rise in patients who subsequently developed clinically evident distant metastases. Taken together, sHER2 monitoring may be useful in monitoring disease progression.

### Future directions

A key question remains to be addressed is whether the sHER2 assay can be used in supplement to IHC/FISH to identify patients who have modestly elevated sHER2 and be responsive to HER2 targeted therapies. This will be helped by the determination of the range of sHER2 value in healthy people by our assay. As we have overcome the initial hurdle for measuring sHER2 due to interference in serum that caused false positive even in normal serum, the normal sHER2 values by our assay could be lower. A study with healthy volunteer serum samples has been planned. In addition, as a high sHER2 preoperative value predicts worse prognosis, sHER2 may be used to further stratify and identify patients who may benefit from newer targeted therapies in future studies. More work is also needed to validate the best clinical use for this assay such as to monitor disease progression for those with high risk tHER2 positive disease.

## Abbreviations

ECD: Extracellular domain
ELISA: Enzyme-linked immunosorbent assay
ER: Estrogen receptor
FISH: Fluorescence in situ histochemistry
HER2: Human epidermal growth factor receptor 2, also called HER2/neu
IHC: Immunohistochemistry
MBB: Magic blocking buffer
PR: Progesterone receptor
sHER2: Soluble HER2
TMB: Tetramethyl benzidine
TNM: Tumor node metastasis
